# Clean with chlorine: peak and short-term occupational exposure to airborne chlorine dioxide during hospital disinfection

**DOI:** 10.1093/annweh/wxag042

**Published:** 2026-06-03

**Authors:** Kasper Solbu, Hans Thore Smedbold

**Affiliations:** Occupational Health Service, Diakonhjemmet, Hartvig Halvorsens vei 2C, Oslo N-0370, Norway; Occupational Health Service, Bærum Municipality, Arnold Haukelands plass 10, Sandvika N-1338, Norway; Department of Public Health and Nursing, Faculty of Medicine and Health Sciences, Norwegian University of Science and Technology, PO Box 8900, Torgarden, Trondheim N-7491, Norway; Department of Occupational Medicine, St.Olav University Hospital, PO Box 3250, Torgarden, Trondheim N-7006, Norway

**Keywords:** disinfection, chlorine dioxide, hospital, occupational exposure, peak exposure, direct reading instruments

## Abstract

This study assessed occupational exposure to airborne chlorine dioxide (ClO_2_) during routine hospital disinfection using a rinsing bottle and textile cloth. Sixty-three personal measurements (>108 h) were collected in operating rooms, patient rooms, and a bed-disinfection unit using direct-reading electrochemical sensors. Exposures were summarized as 8-h time-weighted averages (TWA-8h); fractions of time above 0.1 and 0.3 ppm; and 10 s, 60 s, and 15 min moving averages. TWA-8h exposures were generally below the Norwegian OEL-8h of 0.1 ppm. In contrast, short-term peak exposures were frequent and substantial: all tasks had at least one exceedance of 0.1 ppm on 10 s moving averages (Norwegian recommended reference period), and 78% of measured tasks exceeded 0.1 ppm on 60 s moving averages (German reference period). Peaks above 1.09 ppm (the instrument's upper reporting limit) occurred in 59% of tasks. Peaks above 0.3 ppm were typical across all task types, with the highest short-term exposures during disinfection of patient rooms and operating rooms, especially in periods of intensified activity during infectious outbreaks such as COVID-19. Individual work practices contributed to additional variability. The relatively long response time of the electrochemical sensor (t_90_ up to ∼160 s) likely led to underestimation of true peak magnitudes. Our findings add to emerging evidence that disinfectant use in healthcare is associated with acute respiratory symptoms, and that TWA-8h poorly capture short-term peak exposures. Together, these results underscore the need for revised safety protocols; re-evaluation of exposure limits and the definition of a ceiling value; improved sensor strategies; and clearer operational guidance on how ceiling/STEL metrics (including averaging time) should be applied, to ensure accurate monitoring and assessment for rapidly acting irritants (such as ClO_2_) in real-world hospital settings.

What's Important About This Paper? This study found short-term peak exposures to chlorine dioxide, a disinfectant used in healthcare settings, can frequently exceed exposure benchmarks even when 8-h time-weighted averages remain low. The findings suggest acute, high-intensity episodes may drive risk of adverse health impacts, despite apparent compliance with 8-h occupational exposure limits. The results offer practical evidence to inform exposure monitoring strategies and safer disinfection practices in healthcare, especially during periods of intensified infection control activity.

## Introduction

### Disinfectants

Rising antimicrobial resistance has prompted stronger hygiene practices in healthcare facilities, including stricter cleaning and disinfection routines (Organisation for Economic Co-operation and Development [OECD], 2019). Outbreaks such as the COVID-19 pandemic have further increased disinfection activity, potentially elevating the risk of mucous membrane irritation and asthma among healthcare workers and cleaners (Casey et al. 2017; Hawley et al. 2017).

In Norway, several chemical disinfectants are approved for technical use in hospitals: of 62 registered products, 20 list hydrogen peroxide (H_2_O_2_) as an active ingredient (The Norwegian Medicines Agency 2025). These are used as concentrates, diluted solutions, solids/tablets for dilution, and ready-to-use wipes. Because hydrogen peroxide is corrosive and can irritate/damage the eyes, skin, and respiratory tract (Casey et al. 2017; Hawley et al. 2017), hospitals have sought alternatives perceived as less hazardous. Chlorine dioxide (ClO_2_) has therefore been adopted for several tasks due to its efficacy and perceived lower hazard profile. However, increased use has revealed challenges in keeping inhalation exposure below occupational exposure limits, as demonstrated in this study.

Commercially available hospital disinfection products that contain small amounts (less than 0.3%) of chlorine dioxide are not labeled as hazardous under the EU CLP Regulation (European Parliament and Council 2008). Chlorine dioxide is an oxidizing agent that remains an undissociated gas in solution at near neutral pH and temperatures below 25 °C (Vogt et al. 2010). When released into the air from the solution, it is a potent respiratory irritant and strong oxidant, with the potential to cause eye/nose/throat irritation and headache in exposed workers (ACGIH, 2018). Use of chlorine dioxide for hospital surface disinfection is relatively recent (Noszticzius et al. 2013). During COVID-19, chlorine dioxide-based products were listed as alternatives to ethanol and hydrogen peroxide-based disinfectants (The Norwegian Medicines Agency 2025).

Several studies have linked the use of cleaning and disinfecting agents in healthcare to asthma, work-related respiratory symptoms, and elevated exhaled nitric oxide (Arif and Delclos 2012; Arif et al. 2009; Delclos et al. 2009; Hassel et al. 2022; Mwanga et al. 2023; Svanes et al. 2015). Their findings confirm that chlorine-based products (bleach) and other oxidizing disinfectants are important determinants of airway symptoms among healthcare workers, consistent with an irritant-driven or mixed irritant-sensitizer mechanism.

Due to inadequate hazard labeling, chlorine dioxide disinfectant products have also been implicated in poisonings reported by the US FDA (Lardieri et al. 2021). In 2018, ACGIH revised its recommendation for chlorine dioxide from a TWA of 0.1 ppm with a STEL of 0.3 ppm to a single TLV–Ceiling of 0.1 ppm, based on sub chronic inhalation studies in rodents (NOAEL 0.1 ppm; severe effects and mortality at ≥2.5 to 10 ppm) and epidemiological findings in pulp and paper workers linking acute respiratory outcomes to short duration “gassing” incidents (ACGIH 2018). The rationale was that ClO_2_ exhibits a steep dose response and that acute peaks, rather than cumulative dose, are the primary driver of adverse effects.

Human workplace exposure data for chlorine dioxide remain sparse (ACGIH 2018). In healthcare, one study reported on workers using impregnated wipes for high-level disinfection (Chang et al. 2018). Since the ACGIH reclassified chlorine dioxide from an TWA-8h plus STEL to a single ceiling limit, very few additional quantitative exposure studies have been published (Fontana et al. 2025). A recent investigation in a gnotobiotic mouse research facility used direct-reading monitoring during room and isolator disinfection and found that short-term airborne ClO_2_ concentrations remained below national occupational exposure limits when disinfection protocols, ventilation, and respiratory protection were strictly controlled (Easton et al. 2024). However, that study was conducted in a highly specialized, well-engineered facility and does not necessarily reflect exposure conditions during manual surface disinfection in ordinary hospital wards and corridors. By characterizing ClO_2_ peaks during routine room disinfection in an acute care hospital, our study addresses this gap and extends the limited literature on real-world exposures in general healthcare environments.

### Chlorine dioxide sampling

The traditional method for air sampling of chlorine dioxide uses a midget fritted glass bubbler with a calibrated pump at 0.5 L·min^−1^ (Occupational Safety and Health Administration [OSHA], 1990). To achieve a target concentration of 10% of the 0.1 ppm TLV-C, a sampling time of ∼100 min is required, which is impractical for capturing short-term peaks. Moreover, bubbler sampling is complex in a busy hospital cleaning environment, where frequent bending and reaching are required. Direct-reading electrochemical sensors provide an alternative suited to short-term exposure evaluation, where exposure is expected to occur in peaks rather than at a steady shift-long level. However, such methods are usually less specific than sorbent methods, with subsequent laboratory analysis. It can also exhibit response-time challenges.

### Background and aim

In 2018, the Occupational Health Service of a small community hospital in Oslo was notified that a hydrogen peroxide-based product had been substituted with a ∼0.02% (200 ppm) chlorine dioxide product—a change initially welcomed by workers. Shortly thereafter, exceedances of Norwegian OELs and reports of symptoms (eg headache and respiratory irritation) were noted. An assessment of the new product's use was therefore initiated, to describe exposure to chlorine dioxide during standard manual disinfection of operating rooms, patient beds and pillows, and patient rooms, and to evaluate exposures against Norwegian and international OELs (eg MAK and ACGIH), using readily available electrochemical sensors. Our study adds quantitative short-term exposure data for chlorine dioxide, an oxidizing disinfectant increasingly used in healthcare settings, for which little exposure information has been available.

## Methods

### Sampling location and participation

The study took place at a small community hospital in Oslo (∼1,700 employees; local hospital for ∼140,000 inhabitants), with ∼200 somatic beds and 9 operating rooms. Workers performing disinfection (cleaning personnel and hospital assistants) were recruited in collaboration with line management and the safety representative. Participation was voluntary; all invited personnel agreed to participate. In total, 27 persons participated (19 cleaning personnel; 8 hospital assistants). Measurements were conducted during August to November 2018, November to December 2019, and April to December 2022. The absence of measurements during 2020 to 2021 reflects COVID-19 restrictions.

### Occupational exposure limit values (OEL)

The Norwegian OEL-8h for chlorine dioxide was 0.1 ppm (0.28 mg·m^−3^) at the start of the study, where compliance testing was conducted in accordance with EN 689 (European Committee for Standardization [CEN], 2019). In Norway, for substances with OEL-8h ≤ 1 ppm, an excursion factor of 2 for 15 min (ie 0.3 ppm for chlorine dioxide) is recommended (Norwegian Ministry of Labour and Social Inclusion, 2024). ACGIH recommends a TLV-Ceiling (TLV-C) of 0.1 ppm to minimize respiratory irritation and pulmonary edema (ACGIH, 2018). This 0.1 ppm ceiling value was adopted as a Norwegian OEL in April 2024 (Norwegian Ministry of Labour and Social Inclusion, 2024). However, in November 2025, the Norwegian Labour Inspection Authority (NLIA) published a letter changing the OEL by adopting the Swedish OEL of 0.1 ppm for and a STEL of 0.3 ppm, in addition to initiation of a formal review process of the OEL.

For ceiling values, NLIA recommends a 10 s reference period (Norwegian Labour Inspection Authority [NLIA] 2020), whereas Germany prescribes a 60 s reference period (Committee on Hazardous Substances [AGS] 2010). TLV-C has no defined reference period. NLIA's exposure strategy guideline considers exposure below 10% of the OEL to be “safe”. Germany states an OEL-8h of 0.1 ppm with an excursion factor of 1 for 15 min (Committee on Hazardous Substances [AGS] 2025).

The relationship between highly variable short-term concentrations and the TWA-8h is non-trivial. Modeling and empirical work on intra-workday fluctuations have shown that identical TWA-8h can arise from very different combinations of short-term peaks and troughs, and that short averaging times are more sensitive to peak excursions than long ones (Smith 2022). Therefore, compliance based solely on an TWA-8h may substantially underestimate the importance of brief, high-intensity exposure episodes for agents with a steep concentration–response relationship. In the present study, we therefore report both task-based short-term metrics and full-shift TWAs and interpret our findings primarily against the STEL and ceiling limit for ClO_2_ rather than the historic 8-h value. This approach is consistent with the view that for rapidly acting irritants, peak intensity and duration may be more relevant drivers of risk than cumulative dose over the entire workday.

### Sampling strategy and work tasks

Personal sampling was performed on cleaners during disinfection of operating and patient rooms, and on hospital assistants during manual disinfection of patient beds and pillows.

Measurements were conducted or supervised by an occupational hygienist from the Occupational Health Service. After training, the staff performed several measurements, while the occupational hygienist collected and interpreted all data. After each measurement, staff completed a brief form recording: task type, duration, any symptoms, and approximate disinfectant consumption. The dataset includes disinfection activity (operating room, bed and pillows, patient room), disinfectant usage, sensor period (2 time periods corresponding to sensor replacement), days since sensor replacement, and cleaner ID. The daily total number of disinfections in the department was also recorded to describe workload and routines.

Symptoms were prelisted on the form (none; headache; cough; irritation of skin/nose/throat/eyes; nausea; chest pressure; dizziness; tiredness; unpleasant odor; breathlessness). Disinfectant consumption per task was reported in intervals: < 0.1 L; 0.1 to 0.5 L; 0.5 to 1 L; 1 to 2 L. For the prevalence of reported symptoms, see Table S1 in Supplements.

Measurements occurred in patient rooms (∼20 to 30 m^3^) and in a larger disinfection department with standard ventilation (∼5 air changes per hour, ACh). Operating rooms (100 to 125 m^3^) had higher ventilation rates (20 to 25 ACh).

### Sampling method

Personal exposure was measured using a GasAlertMicro 5 direct-reading gas monitor (BW Technologies by Honeywell, Calgary, Canada) (https://sps.honeywell.com/us/en/products/safety/gas-and-flame-detection/portables/gasalertmicro-5-series, accessed September 1st 2023), equipped with a chlorine dioxide electrochemical sensor (Sensoric ClO2 3E 1 O, City Technology, Bonn, Germany) (City Technology, 2011). The instrument was clipped to the personnel's breathing zone.

The instrument was set to high resolution mode (range 0.01 to 1.09 ppm; 1 s logging). The manufacturer specifies t_90_ < 120 s (typically < 60 s) and long-term sensitivity drift < 10% per 6 mo. The instrument was fitted with a new, externally calibrated sensor before the measurements started (18.06.2018) and after the COVID-19 measurement break (14.09.2021). External calibrations were performed on 05.09.2019 and 22.09.2023.

Concentrations above the instrument's quantification range (“out of limit”, OL) were replaced by the maximum reading (1.09 ppm) for spreadsheet calculations. OL points constituted ∼0.3% of all logged values. No further correction for OL readings was applied.

In December 2021, a CAL 2000 chlorine dioxide generator (Advanced Calibration Designs, AZ, USA) was made available. Before use, the generated ClO_2_ concentration was measured for comparison using the OSHA reference method (Occupational Safety and Health Administration (OSHA), 1990). Since March 2022, it has been regularly used for bump testing. Results were recorded to evaluate sensor drift (Fig. S1). Gas was delivered directly to the sensor via a single gas calibration cap, with 0.5 ppm as the reference test concentration. Instrument response time was tested regularly. During March to December 2022, sensor span drift of approximately ±20% (Fig. S1) and a response time variation of ∼102 to 162 s was observed. Parallel personal sampling using a standard wet-chemical reference method (eg OSHA/NIOSH) was not performed during routine disinfection tasks. The study design required high temporal resolution to characterize second-to-minute peaks across repeated short tasks, and parallel reference sampling was not feasible without interfering with work practice. Instead, the concentration generated for bump testing was verified using the OSHA reference method before use, and repeated bump tests were conducted to track span drift and response time over time.

### Statistical analyses

Data were exported with Honeywell Fleet Manager II (v4.5.60) and processed in Microsoft Excel and RStudio 2026.01.0 + 392. For each measurement, we computed descriptive statistics and 10 s, 60 s, and 15 min moving averages (MA). The number and percentage of OL points per task were recorded. Maximum MA values and fractions exceeding 0.1 and 0.3 ppm were calculated for comparison with OELs.

TWA-8h was estimated assuming 6 repetitions per day for operating/patient room disinfection. For bed and pillow disinfection, each measurement was assumed to represent the day's total exposure. TWA distributions were assumed log-normal. Compliance testing for STEL and OEL-8h followed EN 689 (European Committee for Standardization [CEN] 2019).

A linear mixed effects model (lme4, ver 1.1-37) was used to estimate determinants of ln(TWA-8h) (Eq.1). TWA-8h was log-transformed prior to modeling. Fixed effects included disinfection task type and disinfectant consumption (recorded per task/day; see Table S2). “Cleaner” was included as a random intercept to account for repeated measurements within individuals.


Eq. 1 $$\begin{array}{rl} & \ln\;(\mathrm{TWA}\text{-}8h_{\mathrm{ij}})\;=\;\beta_{0}\;+\beta_{1}Operating\;room_{ij}\\ & \;+\beta_{2}\;Patient\;room_{ij}+\beta_{3}\;\mathrm{Consumptio}n_{\mathrm{ij}}\;+u_{j}+\varepsilon_{ij}, \end{array}$$

where *i* indexes' observations, *j* indexes cleaner, *u_j_* is the random intercept, and ɛ_ij_ is the residual error.

In the mixed-effects model, task type was entered as 2 binary variables (operating room and patient room), using beds and pillows as the reference category. For each observation, the corresponding variable was coded as “1” if the task took place in that setting and “0” otherwise. Thus, the coefficients for the operating room and patient room represent the difference in ln(TWA-8h) relative to beds and pillows, adjusted for disinfectant consumption and repeated measurements within cleaners (random intercept).

Disinfectant consumption was categorized as low (0/0.1) or high (0.5/1). Consumption was not recorded for 8 of the tasks (coded as “-” in the field sheet). In the primary determinants model including consumption, tasks with non-recorded consumption were treated as missing (complete-case analysis; *n* = 55). To assess robustness of task-type effects, we also fitted (i) a model excluding consumption using all observations (*n* = 63) and (ii) a sensitivity model retaining “not recorded” as a separate category.

We evaluated additional sensor-related terms (sensor period and days since sensor replacement) as sensitivity analyses to assess whether residual drift influenced effect estimates; these did not materially change conclusions. Because span drift was tracked through repeated bump testing (Fig. S1), sensor timing variables were not used to “correct” measurements; they were included only as sensitivity terms to confirm that effect estimates were robust to any residual drift.

## Results

### The disinfection work

Cleaning personnel were assigned tasks as needed. Disinfection of operating and patient rooms included window frames; bathrooms and sinks (in patient rooms only); the operating table (in operating rooms only); and general surfaces, such as floors, walls, tables, and chairs. Workers often bent over surfaces at approximately arm's length while pouring solution or wiping with a moist cloth.

For patient rooms, 1 to 10 requisitions (ie logged cleaning/disinfection work orders in the hospital system) were registered per day (median 3.5), totaling 126 requisitions, of which 23% were measured. Disinfection of operating rooms was typically performed 3 to 4 times per week.

During disinfection, the solution was poured directly from the original 1 L bottle through a ∼2 mm opening. For floors, pouring was typically from hip height; for other surfaces, pouring occurred closer to the breathing zone. A freshly opened bottle was used each day. Disinfectant consumption was recorded for 57/63 (90%) periods: 10 reported < 0.1 L; 10 reported 0.1 to 0.5 L; 33 reported 0.5 to 1 L; 2 reported 1 to 2 L (Table S2). Consumption varied more in patient rooms, reflecting differences in room size and room-specific tasks (eg bathrooms). Personal factors and training may also contribute.

Bed and pillow disinfection is generally automated via a tunnel system at the bed central. However, manual disinfection by hospital assistants occurs when the system is unavailable: Beds are elevated to hip height, where assistants work adjacent to the bed, bending while pouring solution or wiping. Manual disinfection targeted bed frames and the waterproof mattress/pillow protective covers—textile linens (sheets/pillowcases) were not treated with chlorine dioxide. Assistants are also allocated to other duties, therefore the measured times ranged 47 to 421 min (Table 1).

**Table 1:** Exposure levels of chlorine dioxide in personal samples during hospital disinfection activities (*n* = 63).

Statistics	Unit	Operating rooms		Beds and pillows		Patient rooms	
		Median	Min.-Max.	Median	Min.-Max.	Median	Min.-Max.
Task							
Number of measurements	(*n*)	17	…	17	…	29	…
Sampling time	min	30	14–49	342	47–421	30	18–45
Exposure (task average)	ppm	0.10 (1.6) ^a^	0.04–0.29	0.02 (1.9) ^a^	0.01–0.06	0.11 (2.0) ^a^	0.03–0.33
15 min max MA^d^	ppm	0.13	0.03–0.48	0.18	0.07–0.40	0.23	0.04–0.48
60 s max MA^d^	ppm	0.30	0.12–0.88	0.65	0.33–1.04	0.43	0.11–0.96
10 s max MA^d^	ppm	0.59	0.19-OL	1.07	0.47-OL	0.73	0.12-OL
Exceedance fractions (task)							
15 min MA^e^ > 0.1 ppm	%	35	0–77	7	0–30	51	0–105 ^c^
15 min MA^e^ > 0.3 ppm	%	0	0–39	0	0–4	0	0–65
60 s MA^e^ > 0.1 ppm	%	36	7–61	7	1–16	42	3–80
10 s MA^e^ > 0.1 ppm	%	35	7–61	6	1–15	42	3–80
Sampling time > OL^f^	%	0	0–3.2	0.2	0–1.4	0	0–3.8
Estimated full-shift exposure							
Duration	min	180	84–294	342	47–421	180	108–270
TWA-8h	ppm	0.03 (1.7) ^a, b^	0.01–0.09 ^b^	0.01 (2.1) ^a^	<0.01–0.03	0.04 (2.2) ^a, b^	0.01–0.11 ^b^
TWA-8h (UTL_0.95, 0.70_)	ppm	0.10 ^b^	…	0.05	…	0.17 ^b^	…

^a^Geometric mean (GM) and geometric standard deviation (GSD) (in brackets).

^b^TWA-8h calculation is based on 6 repetitions per day.

^c^Time for moving average to reach zero exceeds the task duration.

^d^Maximum moving average (max MA) during the measured period.

^e^Moving average (MA).

^f^OL = concentrations above 1.09 ppm recorded as “out of limit”.

Both cleaners and assistants typically worked at approximately arm's length from the applied solution. For cleaners, work tasks were measured; for assistants, the work shift at the bed central was measured. Assistant shifts were eligible for measurement only on days when at least one manual chlorine dioxide disinfection of a bed/pillow occurred. Measurements, therefore, captured both intermittent disinfection episodes and other bed-central duties performed during the same shift.

### Measured chlorine dioxide exposure

Breathing zone concentrations were measured during disinfection of operating rooms (*n* = 17), beds and pillows (*n* = 17), and patient rooms (*n* = 29). Measurement durations ranged from 15 min to 7 h (Table 1). Operating and patient room tasks generally lasted less than 1 h; bed and pillow sessions were typically more than 2 h due to repeated tasks.

The 2 measurements at the bed central with the shortest durations (46 and 88 min) were due to short duty at the bed central for the hospital assistants these days. For the hospital assistants at the bed central, all disinfection tasks during the workday were measured. In contrast, for the cleaning personnel, only one of the (up to 6) disinfection tasks was measured.

**Figure 1:**
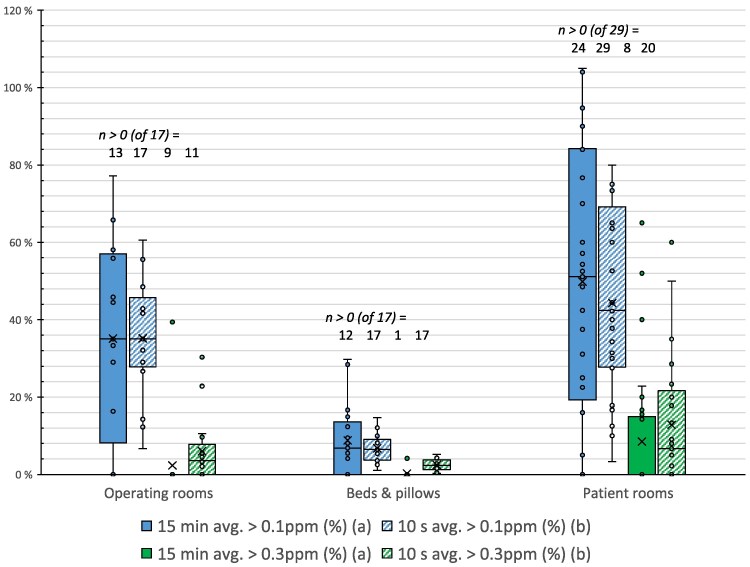
Fraction of task time with MA above 0.1 and 0.3 ppm; for 15 min (solid) and 10 s (striped); for operating rooms (single task), beds & pillows (day activity) and patient rooms (single task) (*n* = 63).

Nearly half of the participants (*n* = 12) were measured once, while the remainder (*n* = 15) were measured 2 to 8 times. Results are summarized by activity in Table 1 and Fig. 1. A sequence plot is provided in Fig. S2, showing no significant systematic changes over time.

The highest task average measured was 0.33 ppm (patient rooms), while the lowest typical task average occurred in bed and pillow disinfection (∼0.01 ppm) (Table 1). Maximum 10 s, 60 s, and 15 min MA values during tasks reached > 1.09, 0.96, and 0.48 ppm, respectively, across activities. The values were in the same range across the 3 types of disinfection tasks. The 15 min MA exceeded 0.1 and 0.3 ppm in 78% and 16% of the measured tasks, respectively. Peaks above 1.09 ppm (the instrument's upper reporting limit; recorded as OL) occurred in 59% of tasks (ie ≥1 OL point per task). Because OL values were censored at 1.09 ppm and the sensor response is finite, short-term MA and TWA estimates should be interpreted as conservative (lower-bound) estimates of true peak-related exposure.

All estimated TWA-8h values were ≤ 0.11 ppm, assuming 6 repetitions for operating room or patient room tasks. The overall geometric mean TWA-8h was 0.03 ppm (GSD 2.4). By activity, GMs (GSDs) were 0.03 (1.7) for operating rooms, 0.01 (2.1) for beds and pillows, and 0.04 (2.2) for patient rooms. Estimated UTL _0.95,0.70_ values were 0.10, 0.05, and 0.17 ppm, respectively (Table 1). This indicates that, with 6 operating/patient room disinfections per day, an OEL-8h of 0.1 ppm is likely to be exceeded (UTL_0.95,0.70_ ≥ 0.1 ppm). For these, the fraction of logged time above OL was as high as 3.8% (patient rooms); for beds and pillows, the maximum %OL was 1.4%.

Task type was strongly associated with exposure in the linear mixed-effects model (Table 2), whereas disinfectant consumption was not. Sensitivity analyses that included all tasks and retained a “not recorded” consumption category yielded essentially unchanged task-type coefficients (Table S3).

**Table 2:** Determinants of chlorine dioxide exposure (linear mixed-effects model).

Determinant	Comparison/coding	Estimate (β)	95% cI (β)	*P*-value	Ratio $e^{\beta }$	95% cI (ratio)
Task type	Operating room versus beds and pillows (as reference category)	1.19	0.54–1.89	0.002	3.29	1.71–6.59
…	Patient room versus beds and pillows (as reference category)	1.36	0.77–2.00	<0.001	3.91	2.15–7.34
Disinfectant consumption	High (0.5/1) versus low (0/0.1)	0.23	−0.20–0.67	0.28	1.26	0.82–1.96

### Exposure patterns

**Figure 2:**
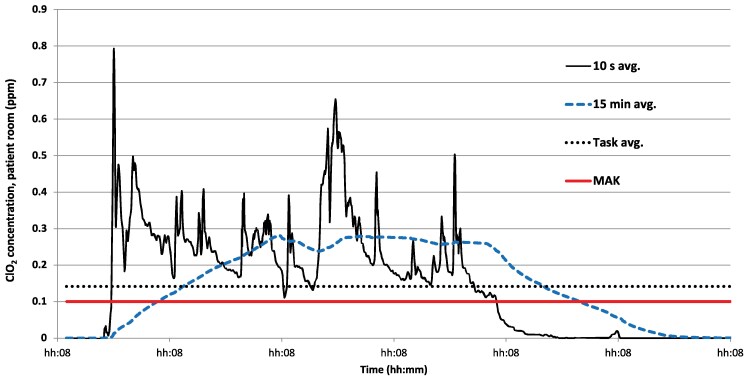
Example of exposure during patient room disinfection (solid line) and 15 min moving average (large, dotted line).

**Figure 3:**
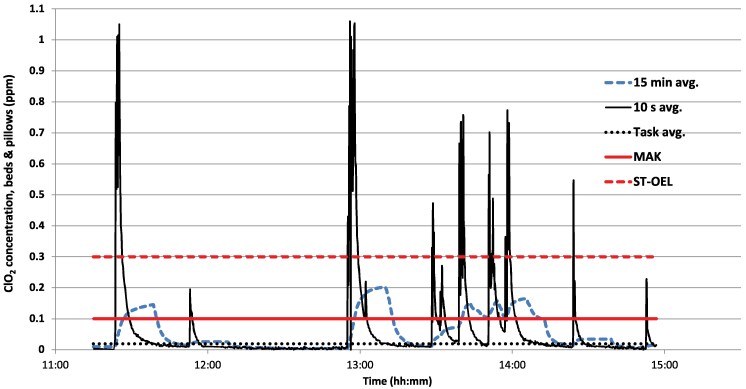
Example of exposure during bed disinfection (solid line) and 15 min moving average (large, dotted line). TWA-8h = 0.003 ppm (dotted line).

Operating and patient room disinfection showed similar patterns: a rapid rise with multiple short peaks. In the illustrative case, the 15 min MA exceeded 0.1 ppm ∼10 min into the task (Fig. 2). In contrast, bed and pillow work displayed numerous peaks throughout the day, with only brief periods when the 15 min MA exceeded 0.1 ppm (Fig. 3).

### Subjective symptoms

Self-reported symptoms included eye/throat irritation, unpleasant odor, tiredness, and headache. Forms were completed for 37/63 activities (59%), and symptoms or unpleasant odor were reported in 16/37 (43%). The odor of chlorine dioxide was commonly observed during operating room and patient room disinfection, consistent with an odor threshold at or below ∼0.1 ppm (AIHA, 2019). No association between symptom occurrence and higher exposure or exposure times was identified. (See Supplement Table S1 for the occurrence of the reported symptoms.)

## Discussion

We assessed personal exposure to airborne chlorine dioxide (ClO_2_) during routine manual hospital disinfection using a rinsing bottle and a textile cloth. While estimated TWA-8h were generally at or below 0.1 ppm, short-term peaks were frequent across all task types, indicating that manual disinfection can generate brief excursions above 0.1 ppm even when shift-average exposure remains low. In our measurements, 15-min moving averages exceeded 0.1 ppm in most tasks, and upper-limit peaks (OL) occurred in more than half of tasks, supporting that the exposure profile is peak-dominated rather than TWA-dominated. Together with previous reports of airway symptoms among healthcare workers exposed to disinfectants, these findings highlight the importance of task-based controls and short-term exposure characterization when low-concentration (∼0.02%) ClO_2_ solutions are used repeatedly in routine practice.

For acute respiratory irritants such as ClO_2_, ceiling/STEL metrics are conceptually more relevant than shift-long averages. However, “peak” definitions vary and are often driven by sampling constraints rather than toxicodynamic considerations (Virji and Kurth 2021). Consistent with methodological recommendations for rapidly varying exposures, we used direct-reading logging and short averaging windows to characterize the frequency and magnitude of short excursions relevant to ceiling/STEL interpretation.

Symptoms or unpleasant odors were reported for a proportion of documented activities (Table S1). However, reporting was incomplete and the study was not designed to quantify exposure–response, so these observations should be interpreted cautiously.

Our findings underscore the importance of task context and individual work practice as determinants of exposure. In mixed-effects analyses (Table 2), task type and between-worker variability accounted for a substantial share of exposure variation, with lower exposures during beds/pillows disinfection than during operating and patient room disinfection. Disinfectant consumption showed no clear association in our data; interpretation is limited by coarse consumption categories and incomplete recording. Moreover, consumption was recorded in broad intervals and may be subject to nondifferential misclassification, which would bias associations toward the null. Consumption was not recorded for 8 tasks; sensitivity analyses retaining a “not recorded” category yielded essentially unchanged task-type estimates (Table S3).

Interpretation of ceiling exceedances must consider the measurement limitations of electrochemical direct-reading sensors. In our setting, system-level response times (t_90_) were on the order of minutes, which can attenuate and broaden short pulses and thus bias peak magnitudes downward when peaks last only seconds. In addition, concentrations above the instrument's upper reporting limit (1.09 ppm) were recorded as OL and replaced by 1.09 ppm, producing conservative lower-bound estimates for short-term moving averages and TWA-8h whenever OL points occur. OL points accounted for a small fraction of logged values (∼0.3%), so their impact on TWA-8h is limited. However, peak magnitude and short-window averages may still be biased downward in tasks with OL excursions. These limitations mainly affect peak characterization; they are less influential for overall task averages and therefore do not change the qualitative conclusion that peaks are frequent.

Finally, bump testing demonstrated span drift over time (Fig. S1), reinforcing the need for systematic bump testing/calibration and improved sensor performance (faster dynamics and wider range) when ceilings/STELs are the compliance target. For compliance frameworks relying on 10 to 60 s windows (Committee on Hazardous Substances (AGS), 2010; Norwegian Labour Inspection Authority (NLIA), 2020), either faster sensors or response-time correction (signal deconvolution) may be considered. Deconvolution can reconstruct peak structure from slow sensors with quantified uncertainty (Dølven et al. 2022).

From a control perspective, our measurements suggest that avoiding ceiling/STEL exceedances during manual disinfection is unlikely without consistent respiratory protection and task-specific engineering/ventilation measures during higher-exposure procedures. This contrasts with findings from a highly engineered gnotobiotic facility (Easton et al. 2024) where controlled protocols, ventilation, and respiratory protection kept concentrations below OELs, underscoring that system design and work practice can materially reduce exposure. The exposure pattern observed here—repeated short peaks above ceiling/STEL limits despite low TWA-8h—also aligns with epidemiological evidence in healthcare linking disinfectant use intensity and frequency to airway outcomes (Mwanga et al. 2023; Fontana et al. 2025).

Our data illustrate the practical challenge of demonstrating compliance with a strict ceiling value in real-world cleaning tasks characterized by pronounced intra-task variability and imperfect control measures. A large proportion of tasks exceeded 0.1 ppm on short averaging windows, and some peaks reached the instrument's upper reporting limit. At the same time, symptom reports were few and incompletely captured, precluding firm exposure–response inference. Together with the known response-time and range limitations of commonly available sensors, these findings highlight that practical ceiling/STEL assessment in such settings may require explicit specification of averaging time (eg 10 s versus 60 s), standardized task-based monitoring protocols, and instrumentation with documented response characteristics aligned with the time-scale of relevant peaks.

Our findings also fit within broader evidence linking healthcare cleaning and disinfection work to respiratory outcomes. A recent systematic review and meta-analysis reported increased odds of respiratory conditions for several disinfectant agents and for general cleaning/disinfection tasks (odds ratios ∼1.4 to 2.3) compared with non-exposed groups (Fontana et al. 2025). Although these studies do not isolate ClO_2_ specifically, they support a prevention strategy centered on selecting less hazardous agents where feasible, improving ventilation and work practices, and ensuring consistent respiratory protection during tasks with predictable peak generation. Emerging cohort evidence also suggests that persistent long-term disinfectant use profiles may be associated with unfavorable asthma trajectories (Pacheco Da Silva et al. 2025), which underscores the relevance of repeated peak exposures over many shifts.

Finally, our study focused on manual application using a rinsing bottle and cloth; the same product can also be used with spray applicators, which are plausibly associated with higher airborne exposure and should be evaluated separately in future work.

## Conclusion

This study shows that the use of a 0.02% chlorine dioxide solution in hospital settings frequently results in exceedances of established occupational exposure limits. Although most TWA-8h remained below the Norwegian OEL-8h of 0.1 ppm under normal conditions, short-term peak exposures exceeded both the STEL of 0.3 ppm and the ceiling value of 0.1 ppm during all types of disinfection tasks (ie at least one short-term excursion above these criteria occurred across task types). Given the limitations of current monitoring technology and the frequent occurrence of such peaks, maintaining exposure below these limits in routine practice will, in most cases, require systematic use of respiratory protection during high-exposure disinfection tasks. Our findings, together with recent evidence on disinfectant-related asthma among cleaners and healthcare workers, support treating manual ClO_2_ disinfection as a task with potential for short-term peak exposure that warrants task-based assessment, improved local ventilation and systematic respiratory protection. More broadly, the results highlight the need for revised safety protocols, re-evaluation of exposure limits and the definition of a ceiling value, improved sensor strategies, and practical guidance on how ceiling limits and short-term metrics should be operationalized in highly variable exposure scenarios.

## Supplementary Material

wxag042_Supplementary_Data

## Data Availability

DataverseNO https://doi.org/10.18710/F7ONJQ.

## Statements

Generative AI (ChatGPT 4 and 5, OpenAI) was used to troubleshoot R scripts and to provide inspiration for rephrasing the manuscript. The authors reviewed and edited all AI-assisted text and take full responsibility for the content. No AI tools were used for data collection, analysis, or interpretation.
