# Supplementary material for: Clean with chlorine: peak and short-term occupational exposure to airborne chlorine dioxide during hospital disinfection
Source: Ann Work Expo Health. 2026 Jun 3;70(5):wxag042. doi: 10.1093/annweh/wxag042 (PMC13232743; doi:10.1093/annweh/wxag042)
Supplement: wxag042_Supplementary_Data [file wxag042_supplementary_data.pdf]

Supplementary materials

Clean with chlorine – Peak and Short-term occupational exposure to airborne chlorine dioxide during hospital disinfection

Kasper Solbu[1,2], Hans Thore Smedbold[3,4]

[1]*Occupational health service, Diakonhjemmet, Hartvig Halvorsens vei 2C, N-0370 Oslo, Norway.*

[2]*Occupational health service, Bærum municipality, Arnold Haukelands plass 10, N-1338 Sandvika, Norway.*

[3]*Department of Public Health and Nursing, Faculty of Medicine and Health Sciences, Norwegian University of Science and Technology. N-7491 Trondheim, Norway.*

[4]*Department of Occupational Medicine, St. Olav University Hospital, PO Box 3250, Torgarden, N-7006 Trondheim, Norway.*

Correspondence to:

Hans Thore Smedbold
Department of Public Health and Nursing,
Faculty of Medicine and Health Sciences,
Norwegian University of Science and Technology,
N-7491 Trondheim, Norway

E-mail: hans.t.smedbold@ntnu.no

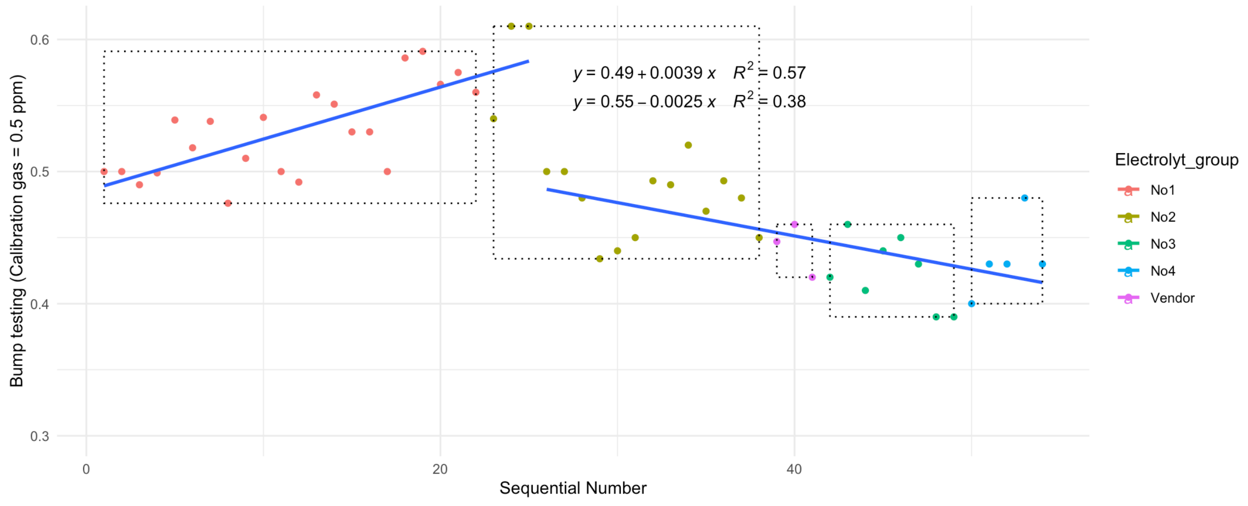

Figure S-1: Bump testing at 0.5 ppm generated a chlorine dioxide atmosphere. The dotted-line boxes comprise samples within the same chlorine dioxide generator electrolyte solution (renewed every 1-2 months).

Alt text Figure S-1: Time-series of repeated bump tests at 0.5 ppm showing variation in sensor response over time, consistent with span drift within approximately ±20%.

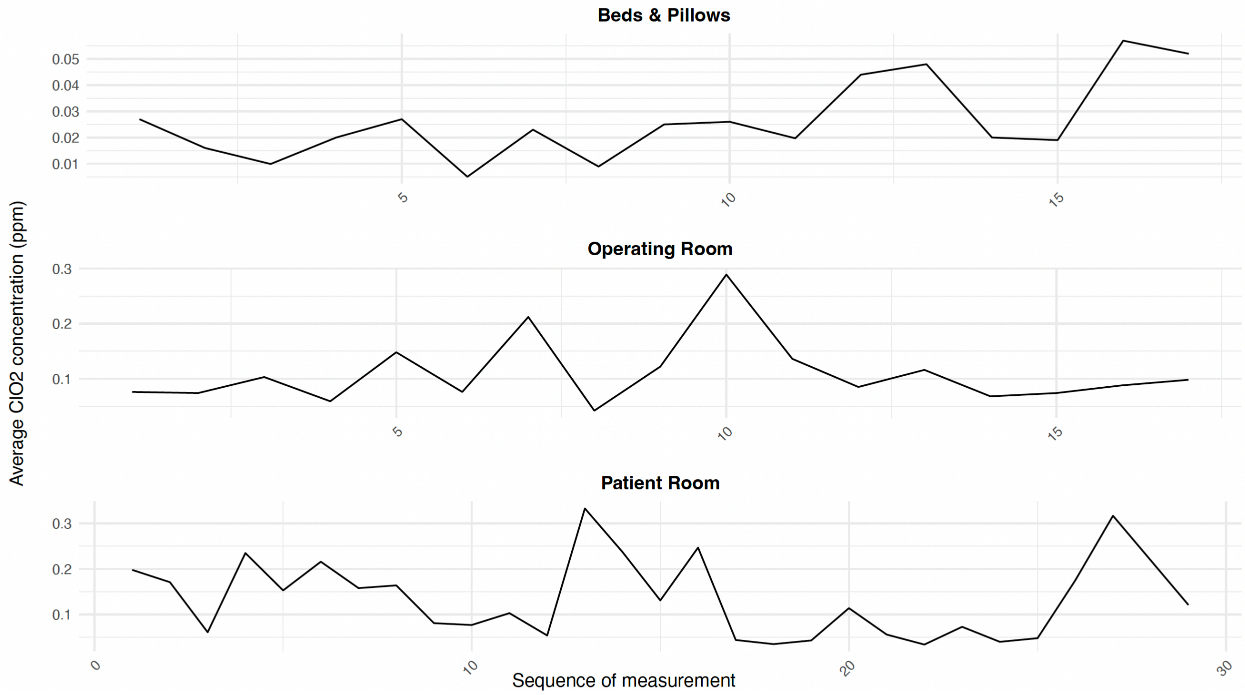

Figure S-2: Sequence plot of chlorine dioxide measurement in disinfection of beds & pillows, operating and patient rooms.

Alt text Figure S-2: Sequence plot of measured chlorine dioxide concentrations across tasks, grouped by task type (beds & pillows, operating rooms, patient rooms), highlighting variability and frequent peaks.

Table S-1: Symptoms reported related to disinfection activities with chlorine dioxide. The number of symptoms is reported by type of activity and measurements (n=63). More than one symptom can be reported per measurement. Figures in bold represent the number of measurements.

Symptoms	Operating Room (n=17)	Beds & Pillows (n=17)	Patient Room (n=29)
**No symptoms experienced**	**3**	**8**	**10**
**Reported symptoms**	**8**	**5**	**3**
-Nasal irritation	6	2	1
-Throat irritation	0	2	1
-Eye irritation	1	0	2
-Skin irritation	1	0	0
-Headache	3	0	0
-Fatigue	2	0	0
-Unpleasant odour	3	5	0
-Other	2	0	0
**Missing**	**6**	**4**	**16**

Table S-2: Consumption of disinfectant reported related to disinfection activities with chlorine dioxide. The volume interval is reported by type of activity and measurements (n=63).

Consumption of disinfectant	Operating Room (n=17)	Beds & Pillows (n=17)	Patient Room (n=29)
Less than 0.1 L	3		7
0.1-0.5 L (half a bottle)	2	2	6
0.5-1 L (full bottle)	6	13	14
1-2 L (1-2 bottles)	1		1
Missing	5	2	1

Table S-3. Determinants of chlorine dioxide exposure (linear mixed-effects model) included measurements with unknown disinfectant consumption (n=63).

Determinant	Comparison / coding	Estimate (β)	95% CI (β)	p-value	Ratio e^{β}	95% CI (ratio)
Task type	Operation room vs Beds & pillows (ref.)	1.13	0.56 - 1.72	<0.001	3.1	1.76 - 5.57
	Patient room vs Beds & pillows (ref.)	1.22	0.68 - 1.78	<0.001	3.4	1.97 - 5.91
Disinfectant consumption	High (0.5/1) vs Low (0/0.1)	0.16	−0.26 - 0.59	0.44	1.18	0.77 - 1.79
	Not recorded vs Low (0/0.1)	−0.19	−0.76 - 0.39	0.51	0.83	0.47 - 1.47
